# The “Y”-shaped Denonvilliers’ fascia and its adjacent relationship with the urogenital fascia based on a male cadaveric anatomical study

**DOI:** 10.1186/s12893-023-01913-y

**Published:** 2023-01-17

**Authors:** Yi Li, Ya-Min Zhao, Yan-Bing Ma, Jin-Song Zhou, Cong Tong, Li-Kun Yan

**Affiliations:** 1 First Department of General Surgery, Shaanxi Provincial People’s Hospital, Xi’an, 710068 Shaanxi China; 2 Department of General Surgery, Shandong Provincial Linyi Jinluo Hospital, Linyi, 276036 Shandong China; 3 Department of Human Anatomy, Histology and Embryology, School of Basic Medical Sciences, Xi’an Jiaotong University Health Science Center, Xi’an, 710061 Shaanxi China

**Keywords:** Denonvilliers’ fascia, Urogenital fascia, Fascia propria of the rectum, Prerectal space

## Abstract

**Background:**

Controversies regarding the anatomical structure of Denonvilliers’ fascia and its relationship with surrounding fasciae have sparked a heated discussion, especially concerning whether Denonvilliers’ fascia is multilayered. This study aimed to expound on the anatomical structure of Denonvilliers’ fascia and its correlation with the peritoneum from the sagittal view and clarify the complex fascial relationship.

**Methods:**

Our study was performed on 20 adult male pelvic specimens fixed in formalin, including 2 entire pelvic specimens and 18 semipelvic specimens. The local adjacent organs and fasciae were dissected, and Denonvilliers’ fascia was observed and removed for histological examination.

**Results:**

Denonvilliers’ fascia was typically single-layered and tough. On the sagittal plane, the peritoneum constituting the peritoneal reflection and Denonvilliers’ fascia formed a “Y” shape. Denonvilliers’ fascia originated from the peritoneal reflection, extended along the ventral side of the seminal vesicles and prostate, continuing caudally; its bilateral sides closely connected to the urogenital fascia (UGF) of the pelvic wall. In addition, histology preliminarily indicated that the basal cell layers of the peritoneum and Denonvilliers’ fascia were continuous and formed a “Y” shape. Furthermore, the basal cells of the two peritonea extended to Denonvilliers’ fascia, creating a fused double-layered structure. Some tiny blood vessels or a network of such vessels extended from the peritoneum to Denonvilliers’ fascia.

**Conclusion:**

Denonvilliers’ fascia, the extension of the peritoneum in the pelvic floor, appears as a single-layered “Y”-shape on the sagittal plane. Our study provides new support for the peritoneal fusion theory. Understanding the anatomical characteristics of Denonvilliers’ fascia and its relationship with the UGF is of guiding significance for inexperienced colorectal surgeons to conduct rectal cancer surgery.

## Introduction

Since the discovery of Denonvilliers’ fascia, its embryological origin, anatomical structure, and association with adjacent fasciae have promoted extensive discussion among anatomical and surgical specialists. There are three mainstream opinions regarding the embryonic origin of Denonvilliers’ fascia, including peritoneal fusion of the embryonic cul-de-sac [1], condensation of embryonic mesenchyme [2], and mechanical pressure [3]. However, no consensus has been reached on this topic [4].

The anatomical features of Denonvilliers' fascia have long been the focus of research by colorectal surgeons since they are closely associated with postoperative recurrence [5] and sexual function after rectal cancer surgery [6]. According to the original description translated by Chapuis et al. [7], Denonvilliers’ fascia is a single layer surrounding the seminal vesicles and prostate. Nevertheless, other scholars have expressed different ideas. Smith [8] and Wesson [2] stated that Denonvilliers’ fascia comprises two layers; however, according to Tobin and Benjamin [1], the posterior layer is the fascia propria of the rectum (FPR). In addition, Ghareeb et al. [9] performed anatomical studies on eighteen cadavers and stated that Denonvilliers’ fascia comprises multiple layers. These different perspectives regarding the components of Denonvilliers’ fascia may confuse inexperienced surgeons, leading them to mistakenly interpret the relationship between Denonvilliers’ fascia and the surrounding anatomy and fail to complete the operation in line with the membrane anatomy theory, especially when the separation is situated in the distal 7 to 10 cm of the rectum at the level of the seminal vesicles in males.

The morphology of Denonvilliers’ fascia is another research focus, and other studies showed a different shape when observing the lateral border. For example, based on findings in cadavers, a previous study demonstrated not only that Denonvilliers’ fascia is a single layer adhering closer to the prostate anteriorly but also that it has no evident lateral border [10]. Therefore, the study concluded that Denonvilliers’ fascia forms an “H”-shaped structure with the adjacent pelvic fascia and perirectal fascia between the prostate and the rectum. In contrast, through 3D reconstruction technology, another study demonstrated that Denonvilliers’ fascia is multilayered and tight in the centre but loose on the bilateral sides and showed Denonvilliers’ fascia on the anatomical dissection of a female foetus, revealing a “Y”-shaped structure [11].

To date, no studies have described the shape of Denonvilliers’ fascia on the sagittal plane or its association with adjacent fasciae by anatomical observation and histology, especially whether Denonvilliers’ fascia continues from the peritoneum. Our previous anatomical study showed that the visceral layer of the urogenital fascia (UGF), which extends along the posterior wall of the urinary bladder and continues down along the seminal vesicles and prostate ventrally, is adjacent to Denonvilliers’ fascia [12].

The controversies regarding the structure of Denonvilliers’ fascia are similar to those surrounding its embryonic origin. None of the current theories provide comprehensive evidence for surgeons to understand Denonvilliers’ fascia and differentiate its surrounding fasciae. Hence, we aimed to improve understanding of Denonvilliers’ facia with an anatomical observational study combined with histology. This new understanding supports the peritoneal fusion theory and may be instrumental in further differentiating the confusing fasciae and achieving the correct operation plane.

## Methods

The entire study was conducted in strict accordance with protocols approved by the Biomedical Ethics Committee of Xi’an Jiaotong University (Ethics Permit Number: 2014-0303). Our study was performed on dissected tissue from 20 formalin-fixed male cadavers provided by the Department of Anthropotomy and Histo-Embryology of Xi’an Jiaotong University Health Science Center. Eighteen of the twenty cadaver samples comprised the hemipelvis with a midsagittal view, and the rest comprised the whole pelvis. The dissections were conducted by two experienced colorectal surgeons from our department, utilizing common surgical instruments (tissue clamps, nontoothed tissue forceps, scalpel, scissors) to avoid complicated techniques for visualization. The informed consent document format was in line with the guidelines of the China Organ Donation Administrative Center.

1. Preparation of whole pelvic samples: The specimen was cut transversely along the plane of the fourth lumbar vertebra and the perineal plane to form the whole pelvis.

Dissection was conducted according to the laparoscopic rectal cancer total mesorectal excision (TME) surgical approach in the retrorectal space to reach the level of the levator ani muscle. Then, separation was continued laterally to the lateral ligaments. Next, the operation was conducted with peritoneal reflection to dissect Denonvilliers’ fascia and enter its anterior space. Additionally, dissection was extended towards the lateral ligaments, which were subsequently cut along the parietal wall of the pelvis to incorporate the lateral and retrorectal space. Special consideration was taken to preserve the continuity of the fascia and maintain its integrity.

2. Preparation of hemipelvic specimens: Eighteen specimens were divided on the sagittal plane with a hacksaw along the midline from the spinous process of the lower lumbar vertebrae to the tip of the coccyx to form a hemipelvic specimen.

From the peritoneal reflection, the peritoneum was separated from the posterior wall of the urinary bladder and the rectum to a certain length. Then, Denonvilliers’ fascia was dissected between the rectum, seminal vesicles, and prostate to maintain its integrity, and its shape and the course of tiny blood vessels were observed.

3. Histology

The 1-cm-wide Denonvilliers’ fascia and the peritoneum connected to both sides were collected from the cadavers and stored in a 10% formalin solution. After routine embedding in paraffin, fixation, sectioning, and staining with haematoxylin–eosin–safranin (HE), the structure of Denonvilliers’ fascia and its extension with the peritoneum were observed under a light microscope.

## Results

### Anatomical observation

**Fig. 1:**
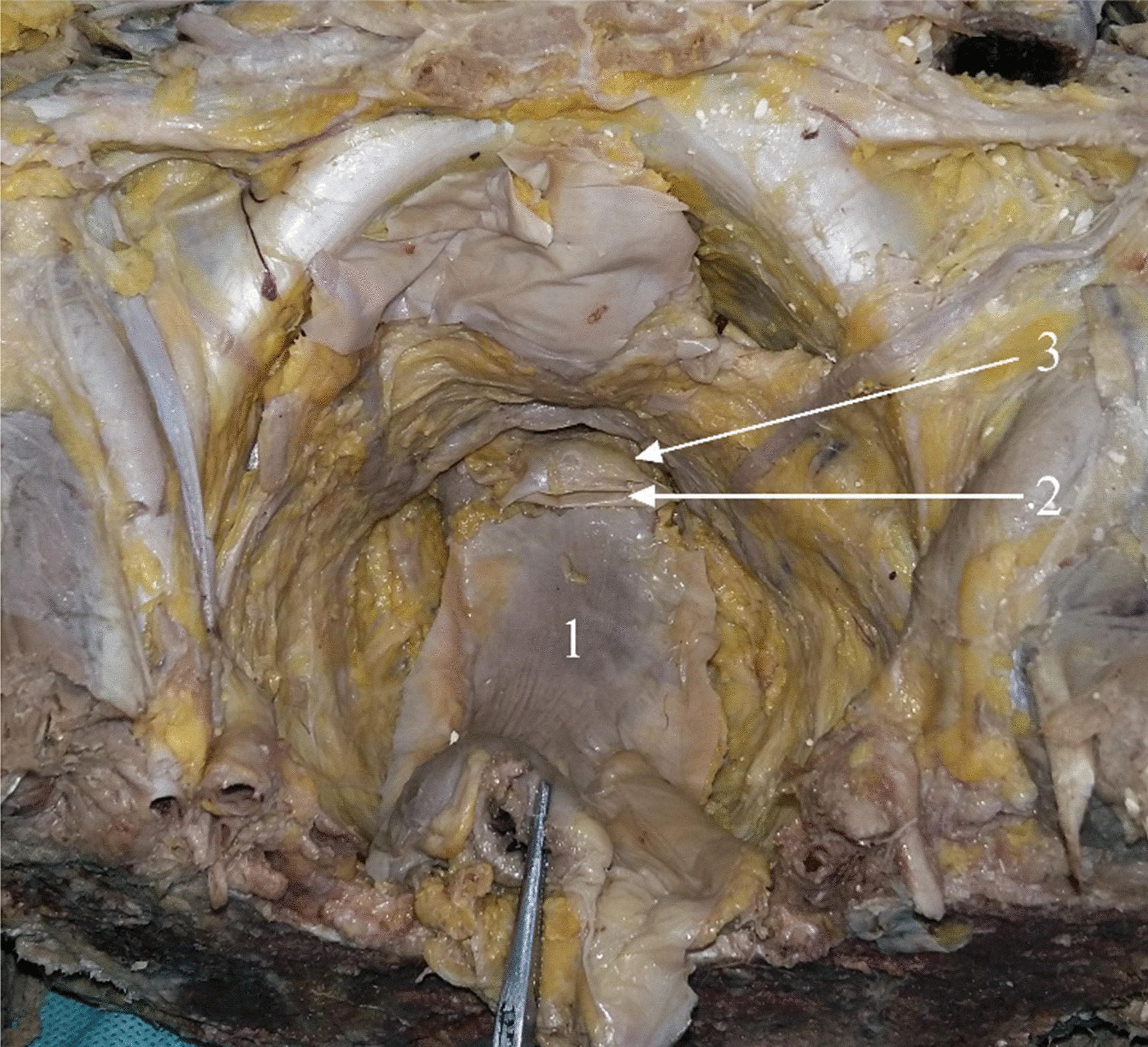
Anatomical observation of Denonvilliers’ fascia on the ventral side. *1* Rectum; *2* Denonvilliers’ fascia; *3* urogenital fascia

All Denonvilliers’ fascia specimens were observed on the ventral side to have a “tongue” shape (Fig. 1).

**Fig. 2:**
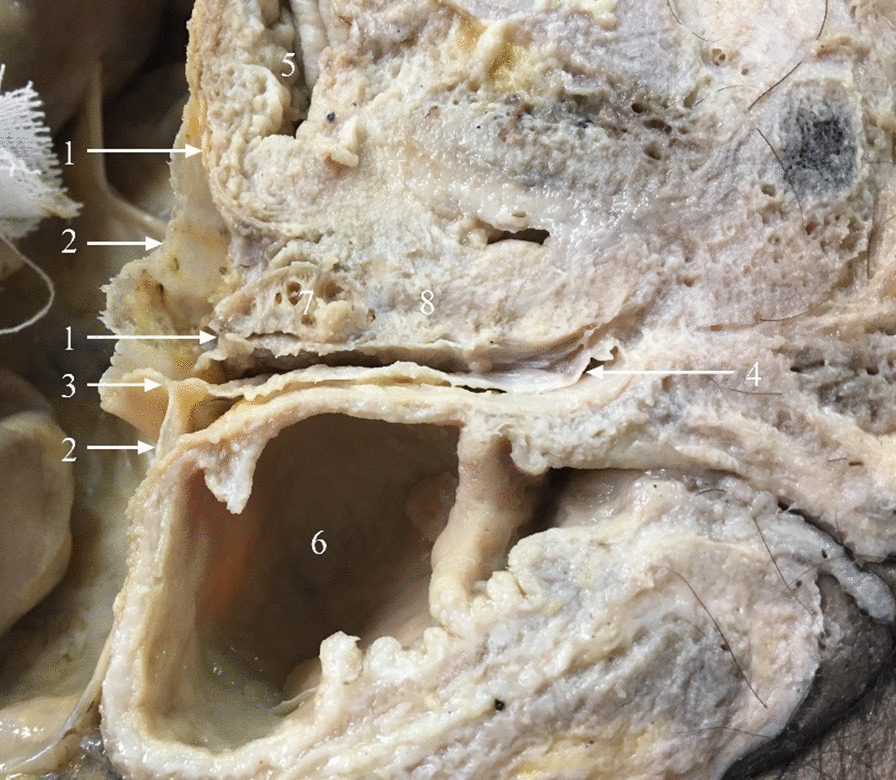
Midsagittal male cadaveric specimens. *1* Visceral layer of the urogenital fascia; *2* peritoneum; *3* peritoneal reflection; *4* Denonvilliers’ fascia; *5* urinary bladder; *6* rectum; *7* seminal vesicles; *8*. prostate

**Fig. 3:**
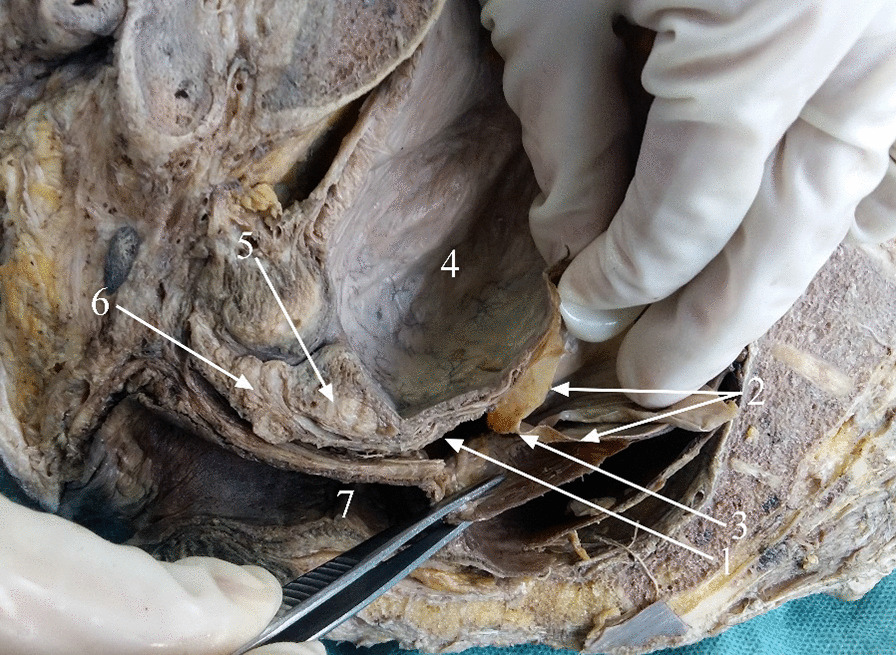
Absence of Denonvilliers’ fascia in the midsagittal plane. *1* Visceral layer of the urogenital fascia; *2* peritoneum; *3* peritoneal reflection; *4* urinary bladder; *5* seminal vesicles; *6* prostate; *7* rectum

On the sagittal plane, the typical Denonvilliers’ fascia formed a “Y” shape. It originated from the deepest point of the peritoneal reflection, extended along the ventral side of the seminal vesicles and prostate, continuing caudally (Fig. 2); its bilateral sides closely connected to the UGF of the pelvic wall (Fig. 1). Denonvilliers’ fascia separated the rectum, seminal vesicles, and prostate; thus, two noncommunicative spaces existed dorsally in the rectum (Fig. 2). In specimens without Denonvilliers’ fascia, these two spaces were absent (Fig. 3). Notably, there were no distinct nerves between the visceral layer of the UGF and Denonvilliers’ fascia.

**Fig. 4:**
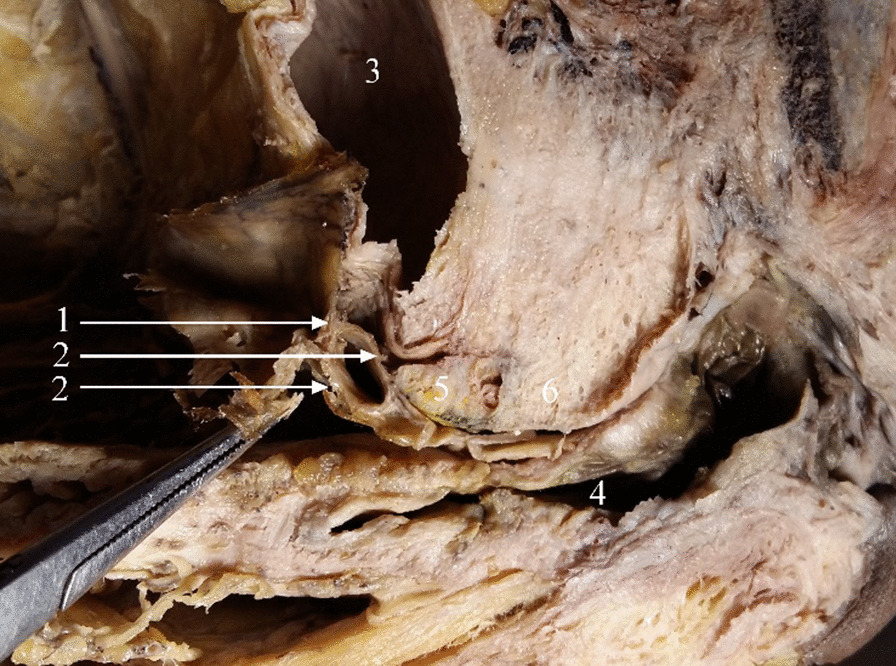
Denonvilliers’ fascia showing two vacuolated layers in this specimen. *1* Peritoneal reflection; *2* two layers of Denonvilliers’ fascia; *3* urinary bladder; *4* rectum; *5* seminal vesicles; *6* prostate

There were two specimens in which Denonvilliers’ fascia was not observed. In one cadaveric specimen, the typical Denonvilliers’ fascia was not found below the peritoneal reflection (Fig. 3). Denonvilliers’ fascia in the other specimen was vacuolated in two layers, resembling a hydrocele (Fig. 4).

**Fig. 5:**
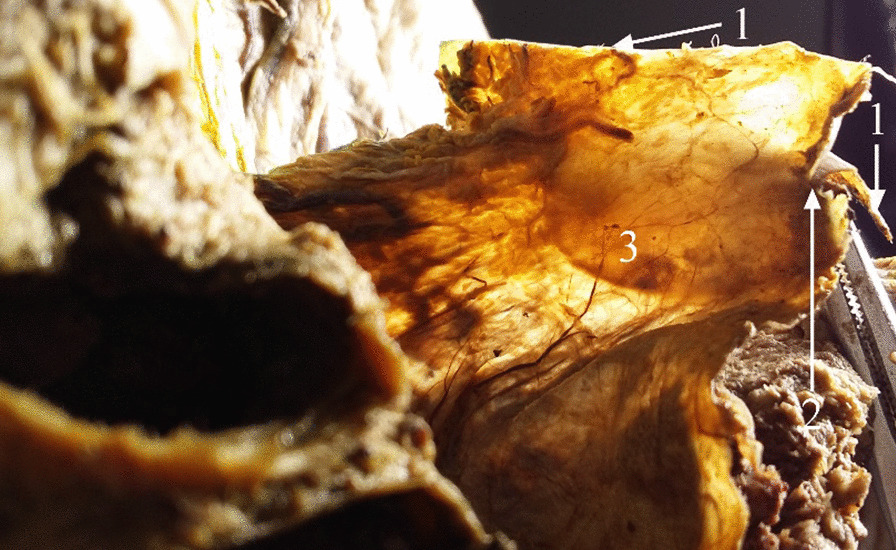
Tiny blood vessels extending from the peritoneum to Denonvilliers’ fascia in cadavers. *1* Peritoneum; *2* peritoneal reflection; *3* Denonvilliers’ fascia

Tiny blood vessels or a network of such vessels extended from the peritoneum to Denonvilliers’ fascia (Fig. 5).

### Histology results

**Fig. 6:**
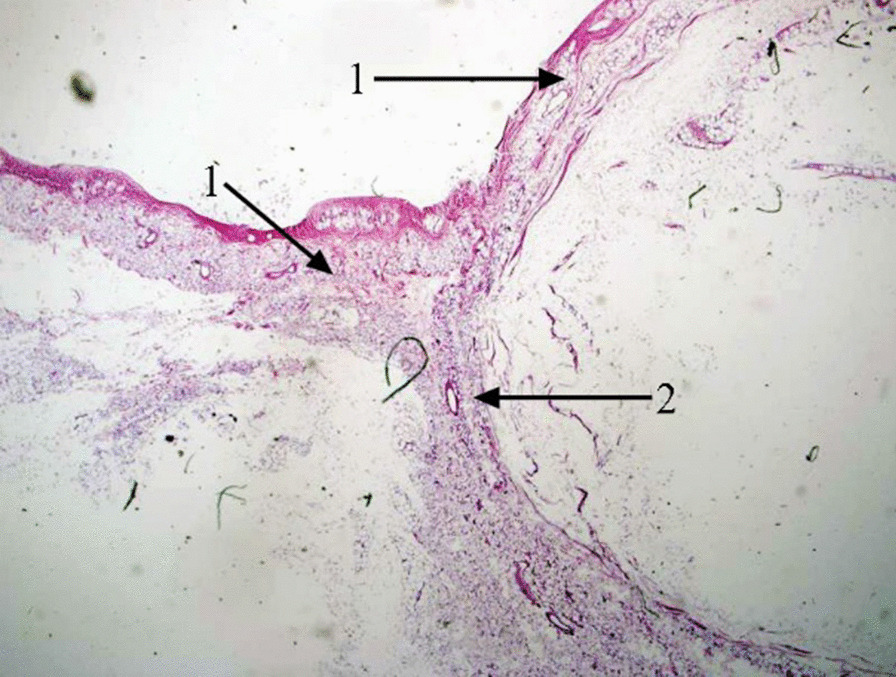
Histological relationship between the peritoneum and Denonvilliers’ fascia. *1* Basal cells of the peritoneum; *2* basal cells of Denonvilliers’ fascia

On the sagittal plane, the peritoneum and Denonvilliers’ fascia formed a “Y”-shaped structure. Under the light microscope, the basal cell layer of the peritoneum appeared continuous with the basal cell layer of Denonvilliers’ fascia and formed a “Y” shape, in which the basal cells layers of two arms of the “Y”-shape (i.e., peritoneum) extended to Denonvilliers’ fascia and fused, forming a bilayered structure (Fig. 6).

## Discussion

Based on our anatomical studies of cadavers, we first propose that Denonvilliers’ fascia constitutes a “Y”-shaped structure on the sagittal plane, which may be conducive for inexperienced colorectal surgeons in discerning its origin, anatomical structure, and correlation with the surrounding fasciae. Viewed on the sagittal pllane, Denonvilliers’ fascia is a monolayered structure that, along with the peritoneum constituting the peritoneal reflection, forms a “Y” shape. Denonvilliers’ fascia originates from the peritoneal reflection, extends caudally between the seminal vesicles and the prostate and rectum. Its bilateral sides closely connect to the UGF of the pelvic wall. In addition, the visceral layer of the UGF, which extends along the posterior wall of the urinary bladder, is located between the seminal vesicles and the prostate and Denonvilliers’ fascia. The histological observations preliminarily indicate that the basal layer of the peritoneum continues with the basal layer of Denonvilliers’ fascia, also forming a “Y”-shaped structure.

Previous studies have extensively discussed whether Denonvilliers’ fascia single, double or multilayered. In a translation of the original description, Chapuis et al. [13] reported that Denonvilliers’ fascia is a single layer enveloping the seminal vesicles and prostate. Other researchers reported that Denonvilliers’ fascia comprises two layers, including the anterior and posterior layers [2, 8]. However, Tobin and Benjamin [1] reported that the posterior layer of Denonviliers’ fascia is the FPR. Likewise, based on a literature review, van Ophoven and Roth [14] supported this view. Notably, Denonvilliers’ fascia has been shown to be a single layer extending from the deepest point of the interprostatorectal peritoneal pouch to the pelvic floor. These anatomical characteristics are consistent with our findings that Denonvilliers’ fascia is derived from the deepest point of the peritoneal reflection. More specifically, Huang et al. [15] performed a cadaver study and proposed that Denonvilliers’ fascia is a monolayered structure situated between the rectum and the bottom of the bladder, seminal vesicles, vas deferens, and prostate. By comparing the results of the related studies, the origin, continuation, and termination points of Denonvilliers’ fascia have common properties, which resemble our results. However, our study elaborates on the anatomical characteristics of Denonvilliers’ fascia in more detail in terms of its morphology, which will help inexperienced colorectal surgeons understand its structure. In addition to the one and two-layer theories regarding Denonvilliers’ fascia, Ghareeb et al. [9] proposed a “multilayer” theory based on an anatomical study. Combining the UGF theory reported in our previous study [12] and the findings of other reports in the literature, we believe that the first layer of “Denonvilliers’ layer” reported by Ghareeb et al. [9] is the true Denonvilliers’ fascia and that the second and third layers represent the visceral layer of the UGF. This conclusion was reached because these two layers extend along the posterior wall of the urinary bladder and the ventral side of the seminal vesicles and prostate and densely adhere to Denonvilliers’ fascia at the caudal end of the prostate.

In addition to the anatomical observation that Denonvilliers’ fascia, the peritoneum and peritoneal reflection form a “Y”-shaped structure, the histological study revealed that the tissue origin of Denonvilliers’ fascia is the same as that of the continuation of the peritoneum. Researchers proposed the peritoneal fusion [1], mesenchymal [2], and mechanical pressure theories [3], and the present study supports the peritoneal fusion theory. Our findings provide new objective anatomical and histological evidence supporting this theory. On the one hand, the cadavers’ anatomy revealed that Denonvilliers’ fascia forms a “Y” shape on the sagittal plane, which is derived from the peritoneal reflection and extends between the rectum dorsally and seminal vesicles and prostate ventrally, continues caudally. On the other hand, the histological observations showed that the basal layer of Denonvilliers’ fascia extendes to the peritoneal basal layer, which also forms a “Y”-shaped structure. Thus, Denonvilliers’ fascia basal cells are formed by the continuous fusion of two layers of basal cells derived from the peritoneum. Our results are similar to those of Bertrand et al. [11], who concluded based on the embryological theory of the female foetus that Denonvilliers’ fascia is in continuity with the peritoneal cul-de-sac. It is conceivable that in the early embryonic period, the peritoneum forms an eggshell-like shape at the bottom of the pelvis, and as the rectum and urinary bladder develop, the lowest area of the peritoneum is squeezed from the ventral and dorsal sides to form Denonvilliers’ fascia.

Notably, two specimens lacked Denonvilliers’ fascia, representing anatomical variation. Unlike the previously recognized variations [16], we believe that a lack of Denonvilliers’ fascia may be linked to the peritoneal fusion theory. As there was no peritoneal fusion in the two specimens, Denonvilliers’ fascia was not formed. This result provides additional evidence for the peritoneal fusion theory, which states that the development of the urinary bladder, seminal vesicles, and prostate enveloped by the UGF and the rectum brings about the fusion of the lowest peritoneum to form Denonvilliers’ fascia.

Our results also provide an anatomical basis for comprehending the surgical identification line of Denonvilliers’ fascia and help clarify the complex fascia between the dorsal rectum and ventral seminal vesicles and prostate. The surgical identification line proposed by Huang et al. [15] is the thickened white line at the peritoneal reflection corresponding to the origin of the “Y”-shaped structure of Denonvilliers’ fascia indicated by our results. Inconsistent with our results, a previous study examined Denonvilliers’ fascia histologically in 25 dissected male cadavers and revealed components with multiple layers [16]. According to our anatomical observations, there are three main layers of fascia between the seminal vesicles, the prostate, and the rectum, including the FPR, Denonvilliers’ fascia, and the visceral layer of the UGF. Therefore, we believe that the visceral fascia between the ventral seminal vesicles and prostate ventrally may underlie the multilayer theory regarding Denonvilliers’ fascia.

In addition, the “Y”-shaped Denonvilliers fascia on the sagittal plane may help one discern the space in the anterior rectum. Similar to Lindsey et al. [17], the space between Denonvilliers’ fascia and the FPR may be called the prerectal space, and the space between Denonvilliers’ fascia and the visceral layer of the UGF can be called the pre-Denonvilliers’ fascial space (i.e., the posterior space of the prostate). Given this defined anterior space of the rectum, separation will be conducted in the pre-Denonvilliers’ fascial space when laparoscopic TME is performed above the white line. If dissection is performed below the surgical identification line, surgical access will be gained through the prerectal space so that Denonvilliers’ fascia can be completely preserved, as recommended by Lindsey et al. [18]. Additionally, similar to some experts [18, 19], we believe that Denonvilliers’ fascia is more tightly adhered to the prostate than to the rectum. Unlike previous studies revealing that nerves run on the anterior aspect of Denonvilliers’ fascia [15, 20, 21], we did not observe nerves between the prostate and Denonvilliers’ fascia but did observe nerves between the prostate and the UGF. Although the operating skill regarding the anterior Denonvilliers’ fascia is divergent, the overall principle is to preserve the nerves. When separating the pre-Denonvilliers’ fascial space, it is crucial to preserve the integrity of the UGF to avoid damaging significant nerves. Depending on the location and stage of cancer, it is reasonable to select either of these two spaces as the separation route in mid-low rectal cancer surgery [4, 22]. After all, Denonvilliers’ fascia is a small piece of peritoneal fusion fascia. Of note, to preserve the nerves that run along the dorsal side of the UGF, the peritoneum above the surgical identification line needs to be cut as thin as possible and dissociated distally along the peritoneum to ensure the integrity of the visceral layer of the UGF. Regarding the separating technique, the experience reported by Huang et al. [15] is consistent with ours. Otherwise, the operation may damage the UGF and seminal vesicles and could damage nerves related to postoperative urinary and sexual dysfunction [10, 22].

The present study has three limitations. First, we could not utilize fresh cadavers, which would have completely avoided postmortem changes. Second, the relationship between the lateral border of Denonvilliers' fascia and the fascia of the pelvic wall was not demonstrated. More importantly, the refined relationship of neurovascular bundles to Denonvilliers’ fascia and the UGF needs further confirmation. Finally, although preliminary histological data indicates that the basal layer of Denonvilliers’ fascia and that of the peritoneum extend together, further research is required to provide more elaborate evidence for the origin of Denonvilliers’ fascia.

## Conclusions

The monolayered structure of Denonvilliers’ fascia along with the peritoneum presented a “Y”-shape originating from the peritoneal reflection, extending on the ventral side of the seminal vesicles and prostate, and continuing caudally; its lateral sides closely connect to the UGF of the pelvic wall. Our study provides new evidence for the peritoneal fusion theory. Comprehending the anatomical characteristics of Denonvilliers’ fascia and its relationship with the UGF provides new knowledge for colorectal surgeons that will improve rectal cancer surgery.

## Data Availability

The datasets generated and analyzed during the current study are available from the corresponding author on reasonable request.

## References

[1] Tobin CE, Benjamin JA (1945). Anatomical and surgical restudy of Denonvilliers' fascia. Surg Gynecol Obstet.

[2] Wesson MB (1923). Fasciae of the urogenital triangle. J Am Med Assoc.

[3] Kim JH, Kinugasa Y, Hwang SE, Murakami G, Rodriguez-Vazquez JF, Cho BH (2015). Denonvilliers' fascia revisited. Surg Radiol Anat.

[4] Zhu XM, Yu GY, Zheng NX, Liu HM, Gong HF, Lou Z, Zhang W (2020). Review of Denonvilliers' fascia: the controversies and consensuses. Gastroenterol Rep.

[5] Heald RJ, Moran BJ, Brown G, Daniels IR (2004). Optimal total mesorectal excision for rectal cancer is by dissection in front of Denonvilliers' fascia. Br J Surg.

[6] Wei HB, Fang JF, Zheng ZH, Wei B, Huang JL, Chen TF, Huang Y, Lei PR (2016). Effect of preservation of Denonvilliers' fascia during laparoscopic resection for mid low rectal cancer on protection of male urinary and sexual functions. Medicine.

[7] Chapuis PH, Kaw A, Zhang M, Sinclair G, Bokey L (2016). Rectal mobilization: the place of Denonvilliers' fascia and inconsistencies in the literature. Colorectal Dis.

[8] Smith GE (1908). Studies in the Anatomy of the Pelvis, with Special Reference to the Fasciae and Visceral Supports: Part I. J Anat Physiol.

[9] Ghareeb WM, Wang X, Chi P, Wang W (2020). The 'multilayer' theory of Denonvilliers' fascia: anatomical dissection of cadavers with the aim to improve neurovascular bundle preservation during rectal mobilization. Colorectal Dis.

[10] Kourambas J, Angus DG, Hosking P, Chou ST (1998). A histological study of Denonvilliers' fascia and its relationship to the neurovascular bundle. Br J Urol.

[11] Bertrand MM, Alsaid B, Droupy S, Benoit G, Prudhomme M (2014). Biomechanical origin of the Denonvilliers' fascia. Surg Radiol Anat.

[12] Li Y, Qin C, Yan L, Tong C, Qiu J, Zhao Y, Xiao Y, Wang X (2021). Urogenital fascia anatomy study in the inguinal region of 10 formalin-fixed cadavers: new understanding for laparoscopic inguinal hernia repair. BMC Surg.

[13] Chapuis P, Zhang M, Bokey L (2019). Use the peritoneal reflection to identify the correct avascular plane posterior to Denonvilliers' fascia. Clin Anat.

[14] van Ophoven A, Roth S (1997). The anatomy and embryological origins of the fascia of Denonvilliers: a medico-historical debate. J Urol.

[15] Huang J, Liu J, Fang J, Zeng Z, Wei B, Chen T, Wei H (2020). Identification of the surgical indication line for the Denonvilliers' fascia and its anatomy in patients with rectal cancer. Cancer Commun.

[16] Muraoka K, Hinata N, Morizane S, Honda M, Sejima T, Murakami G, Tewari AK, Takenaka A (2015). Site-dependent and interindividual variations in Denonvilliers' fascia: a histological study using donated elderly male cadavers. BMC Urol.

[17] Lindsey I, Guy RJ, Warren BF, Mortensen NJ (2000). Anatomy of Denonvilliers' fascia and pelvic nerves, impotence, and implications for the colorectal surgeon. Br J Surg.

[18] Lindsey I, Warren BF, Mortensen NJ (2005). Denonvilliers' fascia lies anterior to the fascia propria and rectal dissection plane in total mesorectal excision. Dis Colon Rectum.

[19] Huland H, Noldus J (1999). An easy and safe approach to separating Denonvilliers' fascia from rectum during radical retropubic prostatectomy. J Urol.

[20] Taguchi K, Tsukamoto T, Murakami G (1999). Anatomical studies of the autonomic nervous system in the human pelvis by the whole-mount staining method: left-right communicating nerves between bilateral pelvic plexuses. J Urol.

[21] Sugihara K, Moriya Y, Akasu T, Fujita S (1996). Pelvic autonomic nerve preservation for patients with rectal carcinoma oncologic and functional outcome. Cancer-Am. Cancer Soc..

[22] Liu J, Huang P, Liang Q, Yang X, Zheng Z, Wei H (2019). Preservation of Denonvilliers' fascia for nerve-sparing laparoscopic total mesorectal excision: a neuro histological study. Clin Anat.

